# The presence of senescent peripheral T-cells is negatively correlated to COVID-19 vaccine-induced immunity in cancer patients under 70 years of age

**DOI:** 10.3389/fimmu.2023.1160664

**Published:** 2023-06-02

**Authors:** E. Orillard, L. Spehner, L. Mansi, A. Bouard, A. Falcoz, Q. Lepiller, E. Renaude, JR. Pallandre, A. Vienot, M. Kroemer, C. Borg

**Affiliations:** ^1^ Department of Oncology, University Hospital of Besançon, Besançon, France; ^2^ Bourgogne Franche-Comté University, INSERM, Etablissement Français du Sang Bourgogne Franche-Comté, UMR1098, Interactions Hôte-Greffon-Tumeur/Ingénierie cellulaire et Génique, Besançon, France; ^3^ ITAC Platform, University of Bourgogne Franche-Comté, Besançon, France; ^4^ Methodology and Quality of Life Unit in Oncology, University Hospital of Besançon, Besançon, France; ^5^ Department of Virology, University Hospital of Besançon, Besançon, France; ^6^ Research Unit EA3181, Université de Franche Comté, Besançon, France; ^7^ Department of Pharmacy, University Hospital of Besançon, Besançon, France

**Keywords:** COVID-19, vaccination, predictive biomarker, cancer patient, T lymphocyte (T-cell), senescence

## Abstract

**Purpose:**

Cancer patients are at risk of severe COVID-19 infection, and vaccination is recommended. Nevertheless, we observe a failure of COVID-19 vaccines in this vulnerable population. We hypothesize that senescent peripheral T-cells alter COVID-19 vaccine-induced immunity.

**Methods:**

We performed a monocentric prospective study and enrolled cancer patients and healthy donors before the COVID-19 vaccination. The primary objective was to assess the association of peripheral senescent T-cells (CD28^-^CD57^+^KLRG1^+^) with COVID-19 vaccine-induced immunity.

**Results:**

Eighty cancer patients have been included, with serological and specific T-cell responses evaluated before and at 3 months post-vaccination. Age ≥ 70 years was the principal clinical factor negatively influencing the serological (p=0.035) and specific SARS-CoV-2 T-cell responses (p=0.047). The presence of senescent T-cells was correlated to lower serological (p=0.049) and specific T-cell responses (p=0.009). Our results sustained the definition of a specific cut-off for senescence immune phenotype (SIP) (≥ 5% of CD4 and ≥ 39.5% of CD8 T-cells), which was correlated to a lower serological response induced by COVID-19 vaccination for CD4 and CD8 SIP^high^ (p=0.039 and p=0.049 respectively). While CD4 SIP level had no impact on COVID-19 vaccine efficacy in elderly patients, our results unraveled a possible predictive role for CD4 SIP^high^ T-cell levels in younger cancer patients.

**Conclusions:**

Elderly cancer patients have a poor serological response to vaccination; specific strategies are needed in this population. Also, the presence of a CD4 SIP^high^ affects the serological response in younger patients and seems to be a potential biomarker of no vaccinal response.

## Introduction

During the COVID-19 pandemic, cancer patients were identified as a high-risk population for serious adverse outcomes, including critical symptoms or death (1). Retrospective studies confirmed this, particularly for patients with metastatic diseases (2), recent treatment with chemotherapy (3), or hematological malignancies. Interestingly, chronological age has also been described as a clinical risk factor for poor outcomes during SARS-CoV-2 infection in this population (3, 4). Following the provision of the vaccine against COVID-19 in early 2021, an unprecedented COVID-19 vaccination campaign began, notably in high-priority patients. The International Society of Geriatric Oncology (SIOG) recommends the prioritization of vaccination plans for elderly patients with cancer, as a population with a higher risk of morbidity and mortality from COVID-19 (5).

Vaccination efficacy, measured by the risk ratio of symptomatic SARS-CoV-2 infection for vaccinated individuals, seems to be reduced in vaccinated cancer patients compared to unvaccinated ones, in a retrospective real-world data study (6). Nonetheless, vaccination effectiveness estimates among patients with solid tumors were equal to 66%. This result is worse than the effectiveness of various approved COVID-19 vaccines efficacy in the global population (89.7 to 95%) (7–10), also based on symptomatic COVID-19 reduction. mRNA vaccines induce antigen-specific antibodies and memory cell responses, including T-cell immunity against SARS-CoV-2 (11). However, this immunization tends to be impaired in subgroups of cancer patients, with an observed lower seroconversion compared to non-cancer patients (12). Additionally, even if SARS-CoV-2 specific T-cell responses do not seem to differ from healthy donors, a large heterogeneity of responses is nevertheless observed among cancer patients (13). Furthermore, immunosuppressive cancer therapy as chemotherapy, stem cell transplantation, cell therapy, or anti-CD20 monoclonal antibodies, negatively impacts specific antibody rate and T-cell responses (14, 15). These results show an inadequate immunization determined by the seroconversion rate and SARS-CoV-2 specific T-cell responses in immunocompromised patients. Hence, the prevention of severe COVID-19 infection by vaccine therapy is based on sufficient immunocompetence.

In the elderly population, it has been shown that the COVID-19 vaccine induced lower B and T-mediated immunogenicity than in younger people (16, 17). The concept of immunosenescence has recently been defined, reflecting the age-associated restructuring modifications of the immune system. This refers to a low-grade, chronic inflammation known as inflammaging (18) and an adaptive immunity dysregulation affecting B cells and the antibody response, but also effector functions of CD4 and CD8 T-cells (19–21). Moreover, there is a decrease in naive T-cells and an enhanced pool of memory T-cells, resulting in an oligoclonal repertoire. Phenotypically, senescent T-cells lose the co-stimulatory molecule CD28 and express the natural killer cell-associated marker KLRG-1. CD57 is also used to identify senescent T-cells (22, 23). However, it is unclear whether this subpopulation is associated with the acquisition of a *de novo* vaccine-induced immunity.

Understanding the limitations of effective COVID-19 vaccination is a challenge in populations at risk of complications. A better understanding of the post-vaccine humoral and cellular response in cancer patients would help optimize the vaccine efficacy (24). We hypothesized that the presence of senescent T-cells might impair the COVID-19 vaccine-induced immunity in cancer patients. This study aimed to evaluate the influence of senescent peripheral T-cells on SARS-CoV-2 vaccine-induced immunity in cancer patients.

## Methods

### Patient selection

The CACOV-VAC study (NCT04836793) was a monocentric prospective study, including cancer patients with active treatment in the adjuvant or metastatic setting, patients in surveillance, and healthy donors. The cut-off age of 70 years was chosen according to the ESMO-SIOG definition as the most commonly used cut-off for defining patients as elderly within the field of geriatric oncology (25). Men or women ≥ 18 years of age were eligible for inclusion if they had been eligible for the vaccination against SARS-CoV-2 with mRNA vaccine (Pfizer: BNT162b2; Moderna: mRNA-1273) and did not develop a symptomatic form of COVID-19 within the last 3 months before inclusion. Major exclusion criteria included the reception of a live, attenuated vaccine within 4 weeks before the initiation of treatment or if this vaccination will be required during the study. Here, we report the results of an ancillary study of the CACOV-VAC trial.

### Assessment of outcomes

The primary objective was to evaluate the association between peripheral T-cell senescence with SARS-CoV-2 specific immunity in cancer patients. The secondary objectives were to define a senescence immune phenotype (SIP) in the CD4 and CD8 T-cell populations and to correlate this SIP with SARS-CoV-2-specific vaccine-induced immunity.

#### Synthetic peptides

Peptides covering SARS-CoV-S protein were purchased from Miltenyi Biotec. Peptivator peptide pools consisting of 15-mer sequences with 11 amino acids overlap represent both CD4 and CD8 T-cells, covering the N-terminal S1 domain sequence of the S protein (1-692 aa named SARS-CoV-Prot_S1), consisting in two functional domains: the S1 domain contains the surface binding site to the ACE2 receptor and the S2 subunit mediates membrane fusion.

#### Assessment of spontaneous T-cell responses against SARS-CoV-2 by IFNγ ELISpot assay

**Figure 1:**
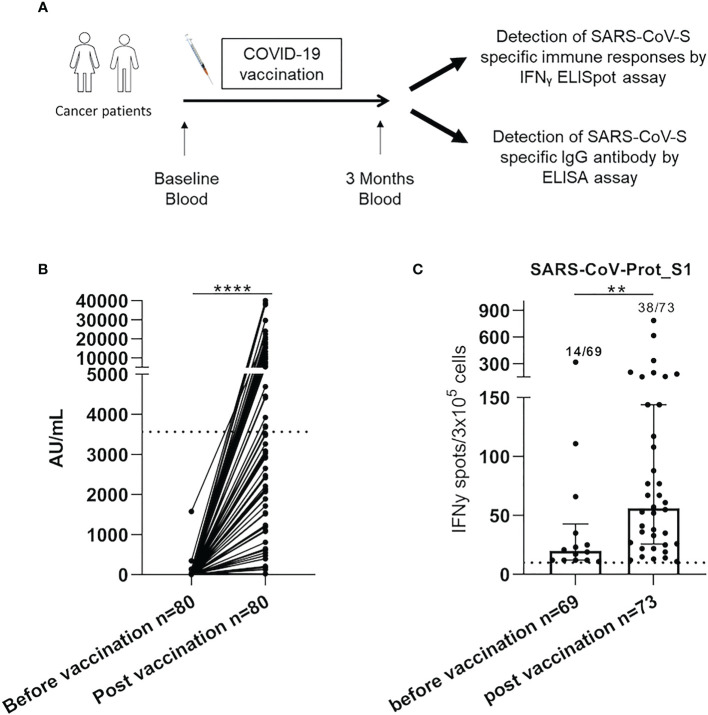
SARS-CoV-2 specific responses were increased after COVID-19 vaccination in cancer patients. **(A)**. PBMC from 80 cancer patients were isolated and analyzed for SARS-CoV-S specific antibody and immune responses by ELISA and IFNγ ELISpot assay, respectively. **(B)** The serological level was analyzed in cancer patients by ELISA assay before and after COVID-19 vaccination in cancer patients (n=80). Graph showing the following responses for each patient. Samples with a result ≥50 (AU/mL) were considered positive according to the manufacturer**’**s instructions. The broken line (3 563 AU/mL) indicated a high serological level. **(C)** PBMC from cancer patients were analyzed for SARS-CoV-S specific T-cell responses by ex vivo IFNγ ELISpot assay before and after COVID-19 vaccination. The intensity of positive SARS-CoV-Prot_S1 specific immune responses in cancer patients. Mann Whitney test, **p<0.01 and ****p<0.0001. Median with interquartile range was indicated on graphs. Responses were considered positive when the IFNγ spot number was ≥10 and the ratio was 2-fold above the background. Only the positive intensities of specific immune responses were indicated.

Specific immune responses were analyzed using Peripheral blood mononuclear cells (PBMC) samples collected before and after the COVID-19 vaccination (**Figure 1A**), by ELISpot IFNγ assay, following the manufacturer’s instructions.

PBMC from cancer patients and healthy donors were isolated by density centrifugation on the Ficoll gradient (Eurobio). PBMC were cryopreserved at a cell density of 8–12 ×106 cells per vial in CryoStor (CS10 and CS5) cell preservation media (Sigma-Aldrich) and were conserved at −196°C for flow cytometry and ELISpot assay analysis. Plasma from cancer patients and healthy donors were isolated by centrifugation and conserved at -80°C for ELISA analysis.

The IFNγ producing SARS-CoV-S specific T-cells responses were quantified by ELISpot assay. For that, 3x105 PBMC per well were cultured in anti-human IFNγ monoclonal antibody in an ELISpot plate with the PepTivators SARS-CoV-Prot_S1 (1μg/mL) in X-Vivo 15 medium (Lonza) for 48 hours at 37°C. Cells cultured with medium alone or Phorbol-12-myristate-13-acetate/Ionomycin (250ng/mL; 10μg/mL, Sigma-Aldrich) were used as negative and positive controls, respectively. All experiments were conducted in duplicates and each result presented is the mean of the duplicates. The IFNγ’s spots were revealed following the manufacturer’s instructions (Diaclone). Estimation of specific T-cell numbers was expressed as spot-forming cells (SFC)/3x105 PBMC and calculated after subtracting negative control values (background). Spot-forming cells were counted using the C.T.L Immunospot system (Cellular technology limited) and assessed with Immunospot 5.0 analyzer software. Responses were considered positive when the IFNγ spot number was ≥10 and the ratio was 2-fold above the background. Only the positive intensities of specific immune responses were represented in this study.

#### IgG Elisa assay

Serological responses were analyzed using serum samples collected before and after the COVID-19 vaccination (**Figure 1A**), by ELISA assay, following the manufacturer’s instructions. Antibodies were detected using the anti-RDB SARS-CoV-S IgG assay on Architect I2000SR (Abbott). Samples with a result ≥50 AU/mL (7.1 BAU/mL) were considered positive according to the manufacturer’s instructions. Antibodies were considered to be at high levels above 3563 AU/mL (506 BAU/ml) for this anti-RBD assay following published data (26). CMV status was determined using the Abbott Architect CMV IgG kit (Abbott) with a positive threshold value of 6.0 AU/mL.

#### Flow cytometry

SIP and immunosuppressive cells were characterized by flow cytometry (**Table S1**). For surface staining, PBMC was washed and stained for 30 min at 4°C in PBS/0.01% BSA and 2mM EDTA with the following Fixable viability Dye (FvD)-eFluor 780 (eBioscience) and antibodies described in **Table S1**, to characterize senescence, memory and M-MDSC populations. For Treg analysis, T-cells were first stained with surface antibodies and fixed and permeabilized for intracellular staining following the manufacturer’s instructions (Foxp3 transcription factor buffer set, BD Bioscience). Samples were directly acquired on a Facs Lyric (BD biosciences) and analyzed with DIVA software.

### Statistical analysis

Continuous parameters were summarized with median and interquartile range (IQR) and compared between subgroups of interest using the Wilcoxon-Mann–Whitney test. Categorical variables were described using absolute and relative frequencies. Proportions were compared using the Chi2 test (or Fisher exact test, if appropriate). The level of significance was set at p < 0.05 for all tests (∗p ≤ 0.05, ∗∗p ≤ 0.01, ∗∗∗p ≤ 0.001, and ∗∗∗∗p ≤ 0.0001). Covariates with p<0.1 in univariable analyses were entered into a multivariable logistic regression model after considering collinearity among variables. Statistical analyses were performed using SAS version 9.4, SPSS 22.0 (IBM, Armonk) considering a significance threshold of 5%.

### Ethical approval

Data were anonymized and the Ethical Review Board on 25/03/2021 approved the study (no. 2021-A00166-35).

## Results

### Heterogeneity of the vaccine-induced SARS-CoV-2 specific immunity in cancer patients

Eighty cancer patients, vaccinated between the 18^th^ of January 2021 and the 25^th^ of May 2021, were included. Three months following vaccination, seventy-eight patients (97.5%) developed a humoral response (>50 AU/mL), and only thirty-five patients (43.8%) had a high serological response (cut-off >3563 AU/mL) (**Figure 1B**). The median intensity of SARS-CoV-Prot_S1 T-cell responses, assessed by ELISpot IFN-γ assay, was significantly enhanced after COVID-19 vaccination (56.0 SFC/3.10^5^ cells (IQR: [25.8-144.0]) vs 20.0 SFC/3.10^5^ cells (IQR: [12.0-42.8]), p=0.006, **Figure 1C**). Moreover, only thirty-eight patients (52.1%) presented positive T-cells responses. These results show the heterogeneity of the COVID-19 vaccine-induced immune responses in cancer patients.

### Age ≥70 years is a clinical factor associated with SARS-CoV-2-specific immunity

Clinical characteristics of patients were presented in **Table 1**, both according to the serological index (high or low), and SARS-CoV-S specific T-cell responses (restricted or without). Patients’ characteristics were similar in both groups, except for age, which was increased in patients with a low serological response compared to patients with a high serological response (66.8 (IQR: [62.7-76.9]) vs 55.0 years (IQR: [49.0-68.7]) p=0.001). The same results were observed for patients without specific T-cell immune responses compared to patients with SARS-CoV-S restricted T-cell responses (68.7 (IQR: [61.7-76.7]) vs 62.5 years (IQR: [47.6-70.3]), p=0.004). These results were confirmed in a multivariate logistic regression analysis (**Tables S2**, **S3**). There was no difference in the humoral or cellular responses according to the stage, or type of treatment, except for cancer location depending on SARS-CoV-S restricted T-cell responses.

**Table 1:** Clinical and immunological parameters of SARS-CoV-2 vaccinated cancer patients according to serology level (cut-off: 3 563 AU/mL) and SARS-CoV-S restricted T-cell responses or non-responders.

	Serology^high^ n=35	Serology^low^ n=45	P-value	SARS-CoV-S restricted T-cell responses n=38	Without SARS-CoV-S T-cell responses n=35	P-value
Age	55.0 [49.0-68.7]	66.8 [62.7-76.9]	p=0.001	62.5 [47.6-70.3]	68.7 [61.7-76.7]	p=0.004
>70 years < 70 years	7 (20.0%) 28 (80.0%)	19 (42.2%) 26 (57.8%)	**p=0.035**	9 (23.7%) 29 (76.3%)	16 (45.7%) 19 (54.3%)	**p=0.048**
Genre						
Male Female	11 (31.4%) 24 (68.6%)	14 (31.1%) 31 (68.9%)	p=0.976	9 (23.7%) 29 (76.3%)	14 (40.0%) 21 (60.0%)	p=0.134
Cancer						
Breast/Gynecological Digestive Lung Other	16 (45.7%) 14 (40.0%) 2 (5.7%) 3 (8.6%)	22 (48.9%) 14 (31.2%) 3 (6.6%) 6 (13.3%)	p=0.847	25 (65.8%) 6 (15.7%) 3 (7.9%) 4 (10.5%)	10 (28.6%) 18 (51.4%) 2 (5.7%) 5 (14.3%)	**p=0.004**
Stage						
Surveillance Adjuvant/Neo-adjuvant L1 metastases	1 (2.9%) 8 (22.8%) 26 (74.3%)	0 (0.0%) 6 (13.3%) 39 (86.7%)	p=0.189	0 (0.0%) 8 (21.0%) 30 (78.9%)	1 (2.9%) 4 (11.4%) 30 (85.7%)	p=0.350
Treatment						
Chemotherapy +/- immunotherapy Immunotherapy Hormonotherapy Targeted therapy Other combination Without treatment	15 (42.8%) 3 (8.6%) 3 (8.6%) 5 (14.3%) 8 (22.9%) 1 (2.8%)	18 (40.0%) 4 (8.9%) 1 (2.2%) 11 (24.4%) 11 (24.4%) 0 (0.0%)	p=0.596	17 (44.7%) 3 (7.9%) 2 (5.3%) 7 (18.4%) 9 (23.7%) 0 (0.0%)	13 (37.1%) 3 (8.6%) 2 (5.7%) 9 (25.7%) 7 (20.0%) 1 (2.9%)	p=0.931
Before vaccination						
CMV Serology level (AU/mL) Missing	92.3 [0.6-250.0] 0	130.9 [0.6-208.9] 1	p=0.963	156.6 [0.9-250.0] 0	123.0 [0.5-245.1] 1	p=0.256
Before vaccination						
Absolute lymphocyte count (G/L) NLR CD4 (%) CD8 (%) CD4/CD8 Missing	1.2 [0.9-1.8] * 2.8 [1.6-3.9] ** 37.5 [29.3-51.1] 18.6 [11.8-30.2] 1.7 [1.2-3.8] 1*/2**	0.9 [0.6-1.5] * 3.3 [2.1-6.8] * 34.9 [24.0-42.1] 25.9 [16.1-31.3] 1.5 [0.9-1.9] 6*	p=0.067 p=0.117 p=0.143 p=0.175 p=0.060	1.1 [0.8-1.5] * 3.1 [2.1-4.3] * 32.9 [29.3-44.0] 22.2 [14.4-32.0] 1.6 [0.9-2.7] 3*	1.0 [0.7-1.7] * 3.0 [1.6-6.1] ** 36.3 [22.1-44.2] 23.1 [13.3-30.7] 1.6 [0.7-2.1] 4*/5**	p=0.409 p=0.984 p=0.569 p=0.765 p=0.721
After vaccination (3 months)						
Absolute lymphocyte count (G/L) NLR missing	1.1 [0.9-1.8] 3.0 [1.7-4.1] 2	1.0 [0.6-1.5] 2.8 [1.8-4.8] 2	p=0.193 p=0.818	1.1 [0.9-1.6] 3.0 [1.8-4.4] 0	1.1 [0.6-1.5] 2.8 [1.8-5.3] 4	p=0.394 p=0.881
Before vaccination						
nTreg (%) eTreg (%) M-MDSC (%) Missing	1.1 [0.9-1.6] * 1.2 [0.6-1.5] * 1.5 [0.6-3.0] 2*/0	0.8 [0.5-1.3] 1.4 [0.9-2.3] 1.4 [0.6-2.5] 4	**p=0.020** p=0.084 p=0.720	0.9 [0.7-1.6] * 1.3 [0.7-1.6] * 1.4 [0.7-2.4] 36 4*/2	1.0 [0.5-1.2] * 1.4 [0.8-2.2] * 1.4 [0.6-2.5] 4*/2	p=0.412 p=0.208 p=0.820
Number of patients with						
SARS-CoV-S restricted T-cell responses without SARS-CoV-S T-cell responses missing	21 (70.0%) 9 (30.0%) 5	17 (39.5%) 26 (60.5%) 2	**p=0.010**	–	–	
High serology level (> 3 563 AU/mL) Low serology level (< 3 563 AU/mL)	- -	- -		21 (55.3%) 17 (44.7%)	9 (25.7%) 26 (74.3%)	**p=0.010**

Medians and proportions were compared between patients with high or low serology levels (3 563 AU/mL) and patients with SARS-CoV-S restricted T-cell responses or non-responders using Wilcoxon–Mann–Whitney and χ^2^ tests (or Fisher’s exact test, if appropriate) respectively. Missing data concerning: (a) absolute lymphocyte count; (b) NLR (c) nTreg and eTreg; (d) M-MDSC. e-Treg, early regulatory T-cells; M-MDSC, monocytic-myeloid derived suppressor cells; NLR, neutrophil-tolymphocyte ratio; n-Treg, natural regulatory T-cells. The data in bold in the p-value column correspond to the significant data.

The biological characteristics of patients were described in **Table 1**, still according to the IgG levels and SARS-CoV-S specific T-cell responses. CD4, CD8, neutrophil-to-lymphocyte ratio, early regulatory T-cells (eTreg), and monocytic-myeloid-derived suppressor cells didn’t influence vaccination efficacy. Patients with a low serology level tended to have a lower CD4/CD8 ratio (1.5 vs 1.7%, p=0.060, confirmed in multivariate analysis; **Table S2**) and natural Treg (nTreg) rates (0.8 vs 1.1%, not confirmed in multivariate analysis). Patients with a high serology level had more SARS-CoV-S T-cell responses compared to patients with a low serology level (21/35 (70.0%) and 17/45 (39.5%) respectively, p=0.010). These results showed that age is a significant clinical factor associated with COVID-19 vaccination responses in cancer patients.

### Circulating senescent T-cells are an immune parameter associated with SARS-CoV-2 specific vaccine-induced immunity and are elevated in elderly cancer patients

**Figure 2:**
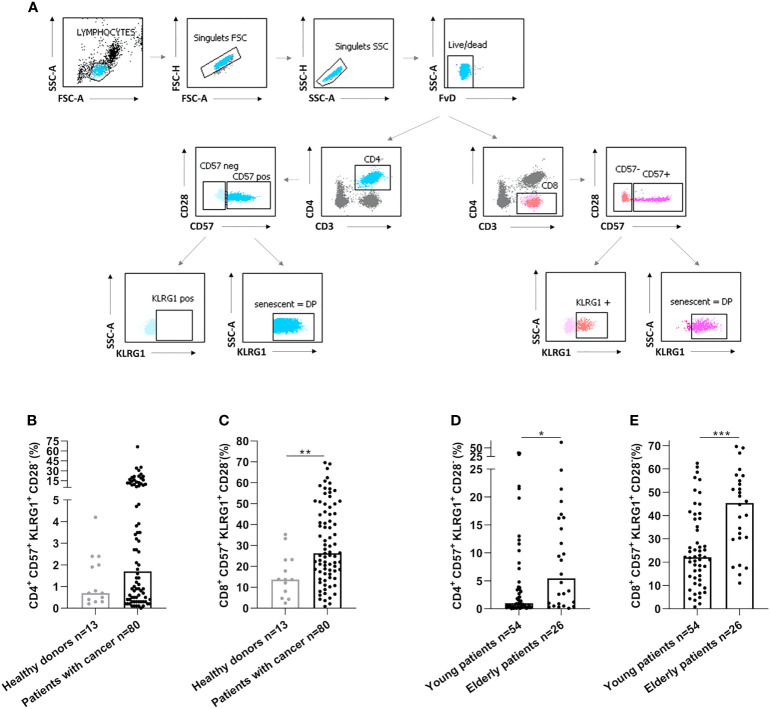
Senescent CD4 and CD8 T-cell populations were increased in elderly cancer patients. **(A)** Gating strategy to analyze immunosenescence T-cells populations by flow cytometry in healthy donors and cancer patients. After the exclusion of doublets and death cells, CD4 and CD8 T-cell populations were selected. The expression of CD57 and KLRG1 were analyzed on CD4 and CD8 T-cells. **(B, C)**. CD57 and KLRG1 expression on CD4 **(B)** or CD8 **(C)** T-cells in healthy donors and cancer patients. **(D, E)**. CD57 and KLRG1 expression on CD4 **(D)** or CD8 **(E)** T-cells according to age in cancer patients. Young patients: < 70 years and elderly patients: ≥ 70 years. Mann Whitney test, where *p<0.05, **p<0.01 and ***p<0.001.

We have then assessed by flow cytometry the senescence phenotype T-cells (CD28^-^CD57^+^KLRG1^+^) before vaccination (**Figure 2A**). Senescent T-cells had effector and late memory phenotypes (**Figure S1**). In cancer patients, we observed an elevation of senescent T-cells compared to healthy donors (p=0.38 and p=0.004, **Figures 2B, C**, for CD4 and CD8 respectively). In addition, senescent T-cells in elderly cancer patients were significantly higher than in younger ones (p=0.025, p=0.0002, **Figures 2D, E**, for CD4 and CD8 respectively). These data show that the rate of circulating senescent T-cells is significantly increased in elderly cancer patients.

**Figure 3:**
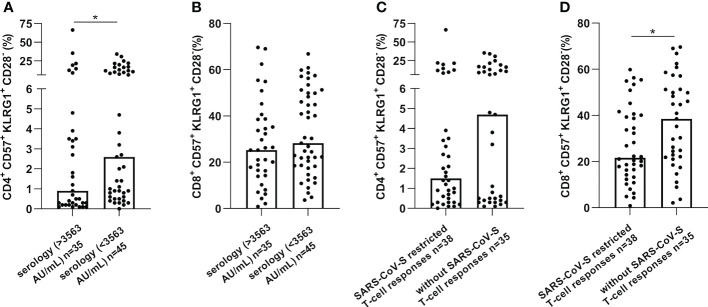
CD57^+^KLRG1^+^ expression on CD4 or CD8 T-cells was increased in cancer patients without immune or serological responses after vaccination against SARS-CoV-2. CD57 and KLRG1 expression were analyzed by flow cytometry in PBMC of cancer patients before vaccination against SARS-CoV-2 (n=80). **(A, B).** Senescence markers expression on CD4 **(A)** or CD8 **(B)** T-cells according to serological level in cancer patients. **(C, D)**. Senescence markers expression on CD4 **(C)** or CD8 **(D)** T-cells according to SARS-CoV-S specific immune responses. Mann Whitney test, where *p<0.05.

Furthermore, a low serological response was significantly associated with more senescent CD4 T-cells (p=0.041) and the absence of SARS-CoV-S specific T-cell responses was associated with more elevated senescent CD8 T-cells (p=0.020), as presented in **Figure 3**. Altogether, these results suggest that senescent T-cells might represent an immune parameter of particular interest in predicting SARS-CoV-2 vaccination-induced immunity in cancer patients.

### Senescence immune phenotypes are correlated with serological and immune responses.

One of the objectives was to define a threshold to determine whether a patient has a senescence immune phenotype (SIP), based on the serological response (**Figure S2**). Restricted cubic splines were used to investigate the relationship between the percentage of CD4 and CD8 senescent T-cells and the odds ratios to predict high serology (cut-off > 3563 AU/mL). An odd ratio of 1 was used to determine the thresholds to define the SIP. Thresholds of SIP T-cell levels have been defined as ≥ 39.5% for CD8 T-cells. For CD4 T-cells, the upper limit of the confidence interval, corresponding to 5%, was used to be more specific about the prognostic value of senescent T-cells.

Elderly patients had more frequently a high SIP for both CD4 and CD8 T-cells according to the SIP threshold (13/25 (52.0%) vs 13/55 (23.6%), p=0.012 and 15/27 (55.6%) vs 11/53 (20.8%), p=0.002 respectively) (**Table 2**). These results were confirmed in a multivariate logistic regression model (**Tables S4**, **S5**).

**Table 2:** Characteristics of cancer patients vaccinated against SARS-CoV-2 according to high or low levels of CD4 and CD8 senescent immune phenotype (SIP) T-cells.

	CD4 SIP^low^ (<5%) n=55	CD4 SIP^high^ (>5%) n=25	P-value	CD8 SIP^low^ (<39.5%) n=53	CD8 SIP^high^ (>39.5%) n=27	P-value
Age	63.7 [51.8-69.7]	70.4 [61.7-76.4]	p=0.140	61.9 [51.6-68.8]	70.9 [65.2-77.1]	p=0.005
>70 years < 70 years	13 (23.6%) 42 (76.4%)	13 (52.0%) 12 (48.0%)	**p=0.012**	11 (20.8%) 42 (79.2%)	15 (55.6%) 12 (44.4%)	**p=0.002**
Genre						
Male Female	18 (32.7%) 37 (67.3%)	7 (28.0%) 18 (72.0%)	p=0.672	12 (22.6%) 41 (77.4%)	13 (48.1%) 14 (51.9%)	**p=0.020**
Cancer						
Breast/Gynecological Digestive Lung Other	25 (45.4%) 19 (34.5%) 5 (9.1%) 6 (10.9%)	13 (52.0%) 9 (36.0%) 0 (0.0%) 3 (12.0%)	p=0.590	31 (58.5%) 15 (28.3%) 3 (5.6%) 4 (7.6%)	7 (25.9%) 13 (48.1%) 2 (7.4%) 5 (18.5%)	**p=0.032**
Stage						
Surveillance Adjuvant/Neo-adjuvant L1 metastases	0 (0.0%) 13 (23.6%) 42 (76.4%)	1 (4.0%) 1 (4.0%) 23 (92.0%)	**p=0.023**	0 (0.0%) 13 (24.5%) 40 (75.5%)	1 (3.7%) 1 (3.7%) 25 (92.6%)	**p=0.012**
Treatment						
Chemotherapy +/- immunotherapy Immunotherapy Hormonotherapy Targeted therapy Other combination Without treatment	24 (37.6%) 6 (7.6%) 3 (7.6%) 8 (20.8%) 14 (26.4%) 0 (0.0%)	9 (36.0%) 1 (4.0%) 1 (4.0%) 8 (32.0%) 5 (20.0%) 1 (4.0%)	p=0.301	20 (37.6%) 4 (7.6%) 4 (7.6%) 11 (20.8%) 14 (26.4%) 0 (0.0%)	13 (48.2%) 3 (11.1%) 0 (0.0%) 5 (18.5%) 5 (18.5%) 1 (3.7%)	p=0.440
Before vaccination						
CMV Serology level (AU/mL) Missing	1.3 [0.5-202.5] 1	207,8 [130.8-250.0] 0	**p=0.0003**	25.2 [0.5-204.6] 1	200.9 [92.3-250.0] 0	**p=0.004**
Before vaccination						
Absolute lymphocyte count (G/L) NLR CD4 (%) CD8 (%) CD4/CD8 Missing	1.0 [0.8-1.5] * 3.0 [2.0-4.6] ** 37.5 [29.5-47.5] 21.4 [13.5-30.2] 1.7 [1.2-2.9] 3*/4**	1.2 [0.8-1.8] * 3.2 [1.6-5.4] * 30.8 [21.0-39.5] 25.9 [17.9-36.9] 1.2 [0.7-1.8] 4*	p=0.531 p=1.000 **p=0.019** p=0.066 **p=0.011**	1.1 [0.8-1.8] * 2.9 [2.0-4.5] ** 35.9 [29.1-46.8] 18.6 [12.2-24.3] 1.8 [1.2-3.4] 3*/4**	1.1 [0.7-1.4] * 3.5 [2.0-6.1] * 31.5 [20.8-40.5] 28.8 [24.3-33.9] 1.3 [0.6-1.7] 4*	p=0.277 p=0.192 p=0.055 **p=0.002** **p=0.0008**
After vaccination (3 months)						
Absolute lymphocyte count (G/L) NLR Missing	1.1 [0.7-1.4] 2.8 [1.8-4.9] 2	1.2 [0.9-1.8] 2.8 [1.6-3.4] 2	p=0.144 p=0.375	1.3 [0.7-1.8] 2.8 [1.8-4.0] 3	1.1 [0.7-1.2] 3.1 [2.1-5.4] 3	p=0.116 p=0.193
Before vaccination						
nTreg (%) eTreg (%) M-MDSC (%) Missing	1.0 [0.7-1.7] 1.3 [0.8-1.8] 1.5 [0.8-2.5] * 2 (c)/ 1(d)	0.9 [0.3-1.2] 1.1 [0.9-1.8] 1.6 [0.5-4.1] * 4(c) / 3(d)	p=0.121 p=0.919 p=0.905	1.0 [0.6-1.5] 1.1 [0.6-1.7] 1.4 [0.6-2.5] 4(c) / 2(d)	0.9 [0.6-1.4] 1.6 [1.3-2.2] 1.5 [0.6-4.1] 2 (c,d)	p=0.869 **p=0.007** p=0.696
Number of patients with						
Without SARS-CoV-S T- cell responses Missing Low serology level (< 3 563 AU/mL)	19 (39,6%) 7 27 (49.1%)	16 (64.0%) 0 18 (72.0%)	**p=0.048** p=0.056	18 (39.1%) 7 26 (49.1%)	17 (63.0%) 0 19 (70.4%)	**p=0.049** p=0.069

Medians and proportions were compared between patients with or without SIP expression on CD4 or CD8 T-cells using Wilcoxon–Mann–Whitney and χ2 tests (or Fisher’s exact test, if appropriate) respectively. Missing data concerning: (a) absolute lymphocyte count; (b) NLR (c) nTreg and eTreg; (d) M-MDSC. e-Treg, early regulatory T-cells; M-MDSC, monocytic-myeloid-derived suppressor cells; NLR, neutrophil-to-lymphocyte ratio; n-Treg, natural regulatory T-cells. The data in bold in the p-value column correspond to the significant data.

There were some clinical differences between both different groups depending on SIP status (**Table 2**). There were more males with CD8 T-cells in the SIP^high^ group than in the SIP^low^ group (13/27 (48.1%) and 12/53 (22.6%), p=0.020), confirmed in a multivariate logistic regression model (**Table S5**). In addition, cancer location, and stage showed significant differences depending on SIP status (**Table 2**) without confirmation in multivariate analysis (**Tables S4**, **S5**).

According to the SIP status, biological factors differed between different groups. Median CMV IgG level was higher in the CD4 and CD8 SIP^high^ group compared to CD4 and CD8 SIP^low^ (207.8 vs 1.3 AU/mL, p=0.0003 and 200.9 vs 25.2 AU/mL, p=0.004, respectively) (**Table 2**), this is confirmed in a multivariate logistic regression model (**Tables S4**, **S5**). In the CD4 SIP^high^ group, the rate of CD4 (30.6% vs 37.1%, p=0.019), and the CD4/CD8 ratio (1.2 vs 1.7, p=0.011) were lower. In the CD8 SIP^high^ group, the rate of CD8 was higher (28.8% vs 18.6%, p=0.002) and the CD4/CD8 ratio was lower (1.3 vs 1.8, p=0.0008). CD4/CD8 ratio was an immune parameter to predict CD4 and CD8 SIP^high^ in a multivariate logistic regression model (**Tables S4**, **S5**). Additionally, the eTreg rate was higher in the CD8 SIP^high^ group (1.6 vs 1.1, p=0.007).

A CD4 SIP^high^ was associated with low serological responses and the absence of SARS-CoV-S T-cell responses (28/55 (50.9%) vs 7/25 (28.0%), p=0.056; and 29/55 (60.4%) vs 9/25 (36.0%), p=0.048, respectively). Also, CD8 SIP^high^ was associated with few SARS-CoV-S T-cell responses (28/53 (60.9%) vs 10/27 (37.1%), p=0.049) (**Table 2**).

Thus, these results suggest that CD4 and CD8 SIP are inversely associated with COVID-19 vaccination immune responses in cancer patients.

### The presence of CD4 SIP affects the post-vaccinal serological response in younger cancer patients

**Figure 4:**
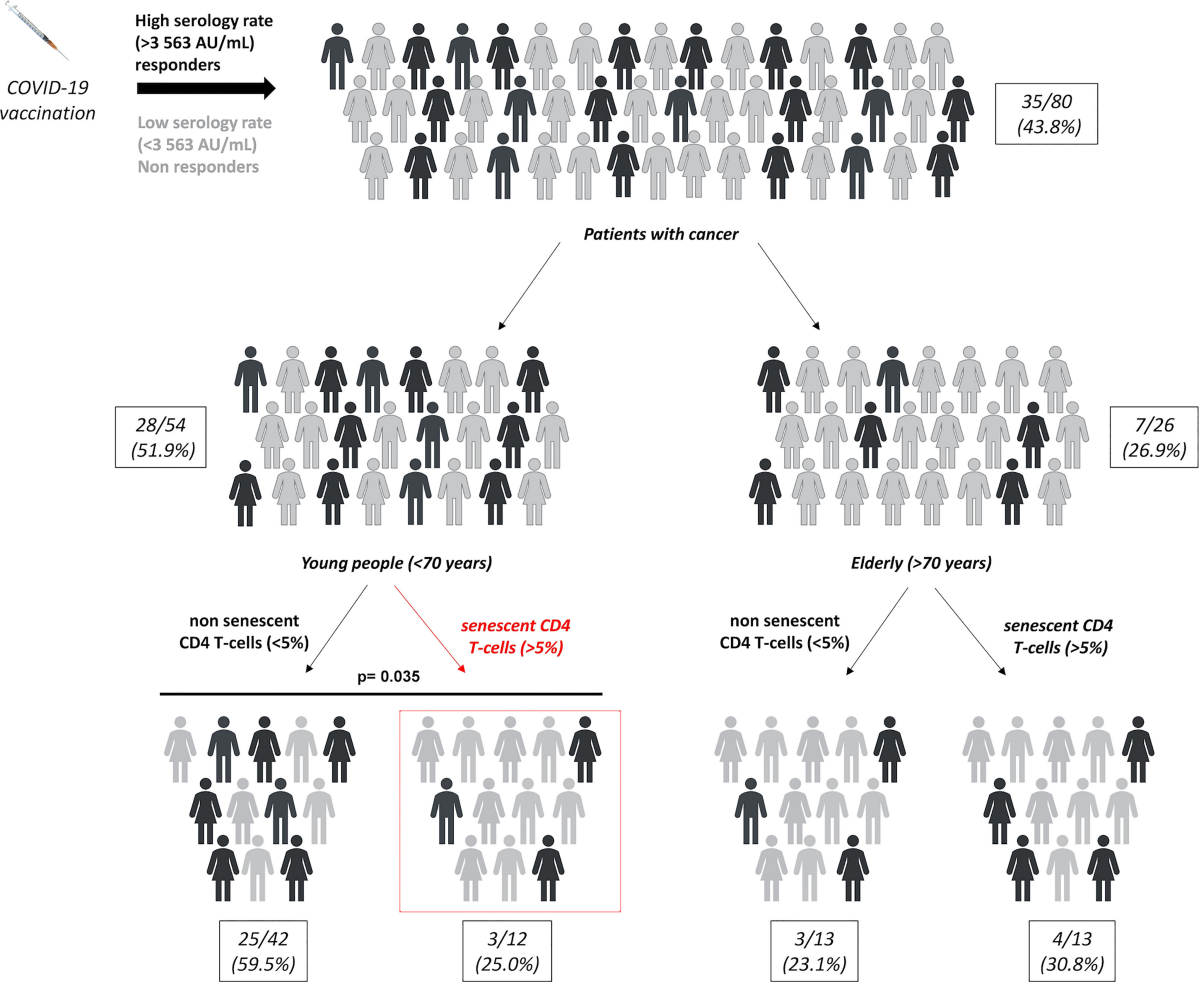
SIP expression on CD4 T-cells affect SARS-CoV-2 response vaccine in younger cancer patients. Repartition of serological responses according to SIP expression on CD4 T-cells and age in cancer patients vaccinated against SARS-CoV-2. Young patients: < 70 years and elderly patients: ≥ 70 years. χ^2^ tests.

Finally, we evaluated the impact of chronological age on the predictive value of CD4 and CD8 SIP T-cells in cancer patients vaccinated by mRNA COVID-19 (**Figure S3**). There was no significant difference according to the patient’s age in terms of T-cell responses depending on the SIP status in cancer patients (**Figures S3A, B**). The same result was observed according to the patient’s age in terms of serological response depending on the CD8 SIP status (**Figure S3C**). Only CD4 SIP status was associated with serological response according to the patient’s age. The proportion of serological response was represented in **Figure 4**, for the eighty cancer patients included in this study. A subgroup of patients was determined according to patients’ age. In the elderly group, no difference in serological response was observed depending on the CD4 SIP status. In the younger group, CD4 SIP^high^ level predicted serological responses induced by COVID-19 vaccination ((3/12 25.0%) vs 25/42 (59.5%), p=0.035) compared to patients with CD4 SIP^low^ (**Figures S3D**, **4**).

Thus, these results suggest that CD4 SIP T-cells could be a potential biomarker to predict SARS-CoV-2 vaccination effectiveness in young cancer patients.

## Discussion

In this study, CD4 and CD8 senescent T-cells, defined by the loss of CD28, and the expression of CD57 and KLRG-1, were negatively associated with serological status and specific T-cell responses, respectively. We identified a senescence immune phenotype (SIP) by the RCS method, corresponding to CD4 senescent T-cells ≥5% of the total CD4 T-cells, and CD8 senescent T-cells ≥39.5% of the total CD8 T-cells. This second set is consistent with the definition given by Ferrara et al. (27). SIP was significantly associated with the absence of SARS-CoV-2-restricted specific responses in cancer patients. Also, a relevant link between CD4 and CD8 SIP^high^ and low serology was observed. According to the patient’s age, SIP expression on CD4 T-cells was associated with lower serological response in the young cancer patient population, but not in the elderly.

Over the last few years, the negative impact of chronological age has been demonstrated on the efficacy of anti-viral vaccines, like in influenza vaccination (28, 29). In the COVID-19 vaccination program, elderly patients are underrepresented in phase II-III randomized clinical trials, limiting the knowledge of vaccine efficacy in this vulnerable population (30). In our study, chronological age is the primary clinical factor affecting COVID-19 vaccine-induced immunity in cancer patients. Given the link between immunosenescence and vaccine efficacy, senescent T-cells have been described to be associated with reduced vaccine responses in many vaccine models (31–33). In the case of COVID-19 vaccination, the impact of general immuno-aging is described in some reviews (34), and the SIOG recommends prioritizing investigations on the impact of aging on vaccine efficacy (5). Recently, Vitallé et al. studied different immune factors in individuals over 60 years of age, compared to younger people, in the specific COVID-19 vaccine immune response. They demonstrated that in vaccinated elderly people, immune defects were associated with lower specific vaccine response. These alterations included the B and T-cells repertoire (35). Therefore, these results imply that the immune system is modified according to chronological age and that this implies a lower vaccine efficacy in older people. In parallel, Huang et al. investigated the causes of poor vaccine response in a healthy population cohort, independently of age. These individuals were shown to have altered specific B cells and less diversity in the specific T cell receptor (TCR) repertoire. The single-cell transcriptomic assessment demonstrated the activation of aging pathways, which may demonstrate that premature aging of lymphocytes contributes to poor vaccine-induced immunity (36). Our results are consistent with these observations, showing chronological age as the main clinical factor of poor response to vaccination, but also that immune-aging of T-cells repertoire impacts the vaccine efficacy. Thus, our study shows that the senescence of T-cells is a compelling potential biomarker of COVID-19 vaccine efficacy.

Independently of chronological age, persistent chronic infection may contribute to poor specific vaccine efficacy. Nicoli et al. demonstrated that CMV seropositivity was associated with a decrease of specific CD4 T-cell responses and antibody responses to vaccination (37). Some works suggest that the link between CMV status and vaccine response is not unique, as it also appears to be related to an expansion of senescent T-cells (32). Moreover, in a recent study in a COVID vaccine model, CMV status has been shown to alter the T repertoire, favoring an effector memory differentiation, without directly impacting vaccine-induced immunity (38). In our study, CMV serological status is not associated with specific B or T-cell response (**Table 1**). However, it is correlated with a SIP (**Table 2**), suggesting that a chronic viral infection could promote T-cell senescence, which could affect vaccine-induced specific responses, as found in previous studies.

Finally, Ferrara et al, in a cohort of patients treated for advanced non-small cell lung cancer, showed that the presence of CD8 SIP impaired the efficacy of immune checkpoint inhibitors (27). In our study, cancer patients seem to have a worse specific vaccine response in the presence of SIP. This can be explained by the presence of KLRG-1 CD8^+^ T-cells is associated with a decreased effector function and clonal expansion of CD8^+^ T-cells (39). Furthermore, TCR diversity is decreased in senescent T-cells. These results suggest that cancer patients exhibit a poorer specific immune response associated with the presence of SIP, regardless of age.

Thus, based on the serological response, cancer patients’ chronological age >70 years negatively impact the SARS-CoV-2 vaccine efficacy. In this elderly population, the only presence of CD4 SIP^high^, described in 50% of this population, is not sufficient to explain the low serological status. Contrarily, in younger cancer patients, more than 20% of this population presents a CD4 SIP^high^ status, which seems to negatively impact the serological response to the SARS-CoV-2 vaccine. Indeed, only 25% of these patients develop a high post-vaccine serological response, corresponding to the serological response of the elderly population in this study. One explanation might be that follicular helper T-cells (Tfh) are required for adequate antibody generation. Indeed, it has been recently demonstrated in mice and humans, that IL-10-producing Tfh are increased with age and impaired humoral responses (40). Additionally, the authors demonstrated that IL-6 is required for the generation of IL-10-producing Tfh, and senescent T-cells have been described to secrete proinflammatory cytokines (39). These results might be explained that CD4 SIP in younger patients is associated with a decreased serological response to the SARS-CoV-2 vaccine compared to younger patients without CD4 SIP.

Therefore, the evaluation of CD4 T-cells senescence appears as a potential biomarker of a high serological response to the SARS-CoV-2 vaccine in the younger cancer patient population. Based on previous data, we suggest that CD4 T-cell senescence can impact vaccine efficacy. In the elderly population, T-cell senescence as analyzed in this study is not the only potential biomarker that can explain the lack of immunological response in this population at risk of complications. The development of investigations to understand the immunosenescence mechanisms and develop specific vaccine strategies in this population is needed. The SIOG recently update recommendations on COVID-19 vaccination to prioritize initial vaccination and vaccine booster in this population at risk and morbimortality from COVID-19 (41). Moreover, from the perspective of the association between senescent T-cells and vaccine efficacy, these results may lead to the development of treatments targeting this population, such as anti-aging drugs (42).

The main strength of this study is that it is currently the first to focus on the association between T-cells immunosenescence and COVID-19 vaccine-induced immunity in cancer patients. Nevertheless, those results need to be validated by a larger prospective cohort.

In conclusion, our study demonstrates that the senescence of CD4 and CD8 T-cells is negatively associated with poor specific COVID-19 vaccine-induced immunity. According to the patient’s age, the presence of CD4 SIP^high^ affects the serological response in younger patients and seems to be an interesting potential predictive biomarker of no vaccinal response. Our results provide new insights into T-cell immunosenescence as a potential circulating biomarker of failure to respond to the COVID-19 vaccine in cancer patients and beyond in an anti-cancer vaccine context.

## Data availability statement

The raw data supporting the conclusions of this article will be made available by the authors, without undue reservation.

## Ethics statement

The studies involving human participants were reviewed and approved by ethical review board on 25/03/2021 (no. 2021-A00166-35). The patients/participants provided their written informed consent to participate in this study.

## Author contributions

LM, MK, and EO conceived and designed the study. LM and MK wrote project administration. AV and AF described the methodology and performed the statistical analysis. LS, ER, AB, and QL realized the investigations. EO, LS, and CB validated the results and interpreted the data. EO and LS wrote the original draft. LM, MK, AF, CB, AV, QL, AB, JP, and ER critically revised the manuscript. LM and MK obtained funding. CB provided supervision. EO realized the submission of the manuscript and is the guarantor of the study and data integrity. All authors approved the final version of the manuscript and agree to be accountable for all aspects of the work.
