# Supplementary material for: The presence of senescent peripheral T-cells is negatively correlated to COVID-19 vaccine-induced immunity in cancer patients under 70 years of age
Source: Front Immunol. 2023 Jun 2;14:1160664. doi: 10.3389/fimmu.2023.1160664 (PMC10272422; doi:10.3389/fimmu.2023.1160664)
Supplement: Supplementary file 1 [file DataSheet_1.docx]

# Supplementary materials





**Figure S1: The effector and late memory phenotypes are associated with senescence T-cells.**

CCR7, CD45RA and CD95 expression were analyzed on conventional and senescence CD4 and CD8 T-cells by flow cytometry in PBMC of volunteer donors (n=13) and cancer patients (n=80) before vaccination against SARS-CoV-2. **A-B.** Memory CD4 T-cells subsets were analyzed in PBMC of volunteer donors (A) or cancer patients (B). **B-C.** Memory CD8 T-cells subsets were analyzed in PBMC of volunteer donors (C) or cancer patients (D). Gray columns represented conventional CD4 T-cells and black columns represented senescence T-cells. Mann Whitney test, where ** p<0.01 and ****p<0.0001.

**Figure S3: Senescent immune phenotype (SIP) expression on CD4 T-cells were associated with non-serological response vaccine in younger cancer patients.** **A-B.** Repartition of SIP expression on CD4 (A) or CD8 (B) T-cells according to SARS-CoV-S specific immune responses in cancer patients vaccinated against SARS-CoV-2 and age **C-D.** Repartition of SIP expression on CD4 (C) or CD8 (D) T-cells according to serological response in cancer patients vaccinated against SARS-CoV-2 and age. Young patients: < 70 years and elderly patients: ≥ 70 years. χ^2^ tests, where * p<0.05.


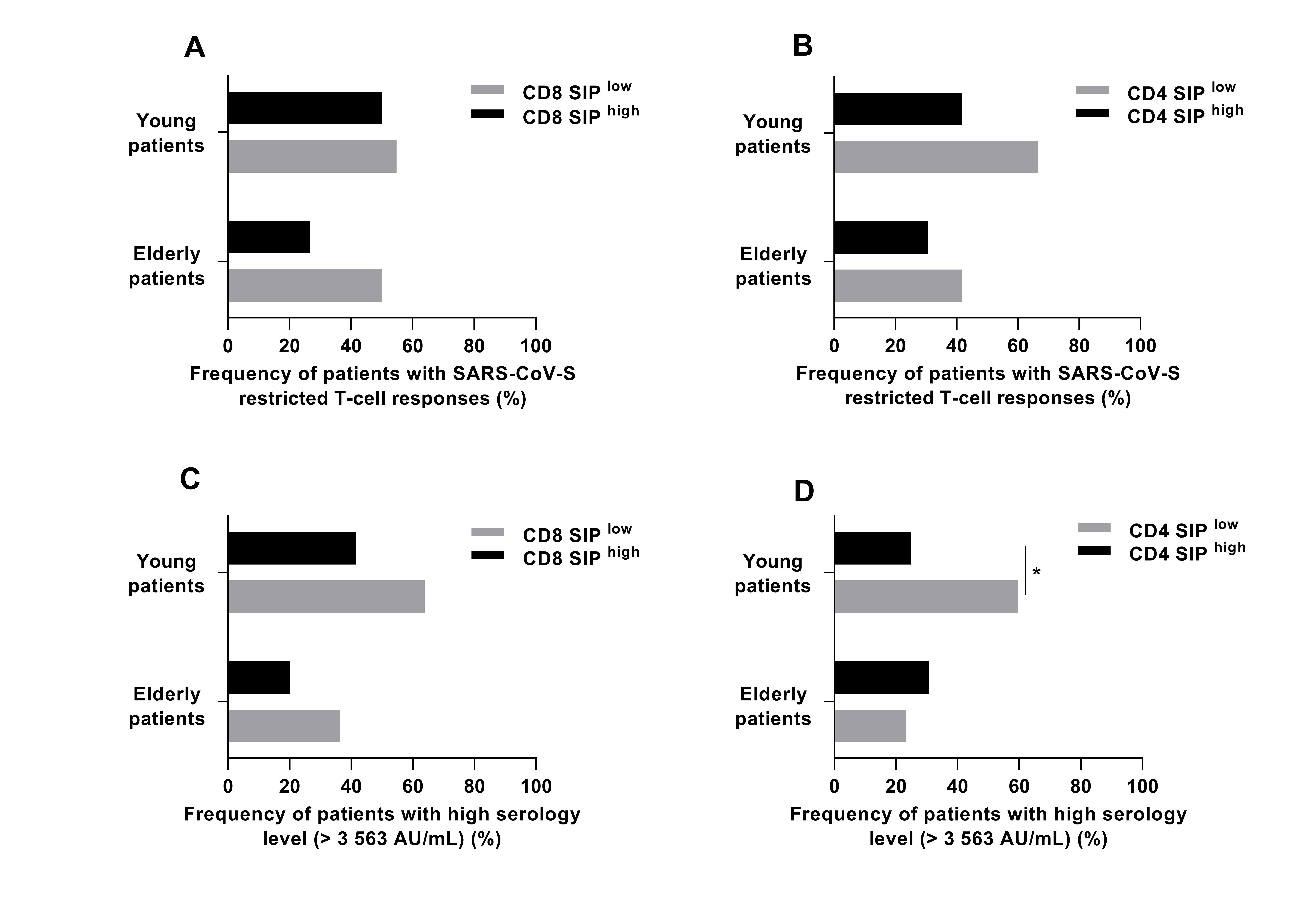

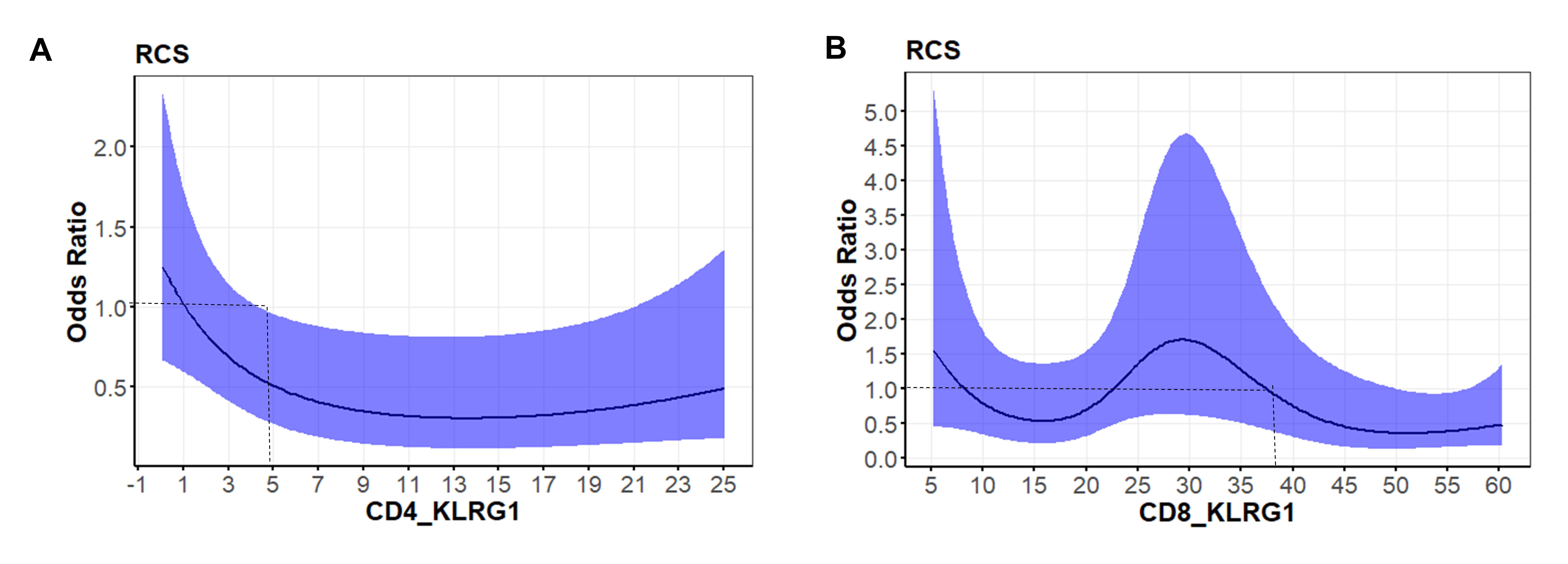


**Figure S2: Determining thresholds for senescent immune phenotype (SIP) on CD4 or CD8 T-cells in cancer patients.** The thresholds of senescent CD4 (**A**) and CD8 (**B**) T-cells were chosen with RCS method, based on serological response.

**Table S1. Description of antibodies using in Flow cytometry**

| **Panels** | **Antibodies** | **Clones** | **Labels** | **Suppliers** |
| --- | --- | --- | --- | --- |
| **Senescence and memory T-cells** | CD3 | HIT3a | BV605 | BD biosciences |
|  | CD4 | RPA-T4 | BV711 | BD biosciences |
|  | KLRG1 | 2F1/KLRG1 | BV421 | Biolegend |
|  | CD28 | CD28-2 | A700 | Biolegend |
|  | CD57 | QA17A04 | BV786 | Biolegend |
|  | CCR7 | 150503 | FITC | Biotechne |
|  | CD95 | DX2 | BB700 | BD biosciences |
|  | CD45RA | HI-100 | APC | BD biosciences |
| **M-MDSC** | Lineage  (CD3, CD56 and CD19) | OKT3, HCD56 and SJ25C1 | Pacific blue | Biolegend |
|  | CD14 | M5E2 | BV605 | BD biosciences |
|  | CD33 | WM53 | BV510 | BD biosciences |
|  | CD11b | ICRF44 | PeCy7 | BD biosciences |
|  | HLA-DR | B-F1 | FITC | Diaclone |
| **Treg cells** | CD3 | HIT3a | BV605 | BD biosciences |
|  | CD4 | RPA-T4 | BV786 | BD biosciences |
|  | CD25 | MA-251 | BV421 | BD biosciences |
|  | CD15s | LSLEX1 | A488 | BD biosciences |
|  | CD45RA | HI-100 | APC | BD biosciences |
|  | Foxp3 | 259D/C7 | PE | BD biosciences |

**Table S2. Multivariate logistic regression model to predict high serology**

|  | **OR** | **95%CI** | **P-value** |
| --- | --- | --- | --- |
| **Age** | 0.913 | 0.869-0.960 | **0.0004** |
| **CD4/CD8** | 1.750 | 1.193-2.566 | **0.0042** |

**Table S3. Multivariate logistic regression model to predict SARS-CoV-S restricted T-cell responses**

|  | **OR** | **95%CI** | **P-value** |
| --- | --- | --- | --- |
| **Age** | 0.944 | 0.901-0.990 | **0.0160** |
| **Cancer (ref=Breast/Gynecological)** |  |  | **0.0265** |
| Digestive vs Breast/Gynecological | 0.156 | 0.046-0.535 | **0.0031** |
| Lung vs Breast/Gynecological | 0.559 | 0.075-4.185 | 0.5715 |
| Other vs Breast/Gynecological | 0.289 | 0.058-1.446 | 0.1308 |

**Table S4. Multivariate logistic regression model to predict CD4 SIP high**

|  | **OR** | **95%CI** | **P-value** |
| --- | --- | --- | --- |
| **Age (≥ 70 vs ref < 70)** | 3.660 | 1.156-11.589 | **0.0273** |
| **CMV serology** | 1.008 | 1.002-1.013 | **0.0086** |
| **CD4/CD8** | 0.597 | 0.355-1.005 | **0.0522** |

**Table S5. Multivariate logistic regression model to predict CD8 SIP high**

|  | **OR** | **95%CI** | **P-value** |
| --- | --- | --- | --- |
| **Age (≥ 70 vs ref < 70)** | 7.557 | 1.891-30.208 | **0.0042** |
| **Sex** | 5.609 | 1.435-21.920 | **0.0132** |
| **CMV serology** | 1.006 | 1.000-1.012 | **0.0562** |
| **CD4/CD8** | 0.347 | 0.164-0.737 | **0.0059** |
